# Integrated Analysis of the Mechanisms of Da-Chai-Hu Decoction in Type 2 Diabetes Mellitus by a Network Pharmacology Approach

**DOI:** 10.1155/2020/9768414

**Published:** 2020-04-28

**Authors:** Beida Ren, Ling Tan, Yiliang Xiong, Wenting Ji, Jie Mu, Yuying Pei, Fafeng Cheng, Xueqian Wang, Qingguo Wang

**Affiliations:** School of Traditional Chinese Medicine, Beijing University of Chinese Medicine, Beijing 100029, China

## Abstract

**Background:**

The incidence of type 2 diabetes mellitus (T2DM) has increased year by year, which not only seriously affects people's quality of life, but also imposes a heavy economic burden on the family, society, and country. Currently, the pathogenesis, diagnosis, and treatment of T2DM are still unclear. Therefore, exploration of a precise multitarget treatment strategy is urgent. Here, we attempt to screen out the active components, effective targets, and functional pathways of therapeutic drugs through network pharmacology with taking advantages of traditional Chinese medicine (TCM) formulas for multitarget holistic treatment of diseases to clarify the potential therapeutic mechanism of TCM formulas and provide a systematic and clear thought for T2DM treatment.

**Methods:**

First, we screened the active components of Da-Chai-Hu Decoction (DCHD) by absorption, distribution, metabolism, excretion, and toxicity (ADME/T) calculation. Second, we predicted and screened the active components of DCHD and its therapeutic targets for T2DM relying on the Traditional Chinese Medicine Systems Pharmacology Analysis Platform (TCMSP database) and Text Mining Tool (GoPubMed database), while using the Database for Annotation, Visualization, and Integrated Discovery (DAVID) to obtain T2DM targets. Third, we constructed a network of the active component-target, target-pathway of DCHD using Cytoscape software (http://cytoscape.org/,ver.3.5.1) and then analyzed gene function, related biological processes, and signal pathways through the DAVID database.

**Results:**

We screened 77 active components from 1278 DCHD components and 116 effective targets from 253 ones. After matching the targets of T2DM, we obtained 38 important targets and 7 core targets were selected through further analysis. Through enrichment analysis, we found that these important targets were mainly involved in many biological processes such as oxidative stress, inflammatory reaction, and apoptosis. After analyzing the relevant pathways, the synthetic pathway for the treatment of T2DM was obtained, which provided a diagnosis-treatment idea for DCHD in the treatment of T2DM.

**Conclusions:**

This article reveals the mechanism of DCHD in the treatment of T2DM related to inflammatory response and apoptosis through network pharmacology, which lays a foundation for further elucidation of drugs effective targets.

## 1. Introduction

Type 2 diabetes mellitus (T2DM), as the most common type of diabetes, refers to metabolic imbalance of glucose, protein, lipid, water, and electrolyte, caused by genetic, environmental, and psychological factors. It is pathophysiologically characterized by a declined ability of insulin to regulate glucose metabolism accompanied by a decrease or relative decrease in insulin secretion due to defects of islet B-cell function. If one's random blood glucose is ≥11.1 mmol/L or fasting blood glucose is ≥7.0 mmol/L, the patient can be diagnosed as diabetes when accompanying with typical symptoms including polydipsia, polyuria, polyphagia, and unexplained weight loss [1]. Diabetes is one of the three major threats to human health. In the later stage, it will lead to the complications such as cardiovascular and cerebrovascular diseases [2], renal injury [3], retinopathy [4], diabetic foot [5], and neurological disease [6], which are the main reasons for its death rate in diabetic patients. Currently, the incidence of diabetes is rising sharply. The number of diabetics worldwide has increased from 108 million in 1980 to 422 million in 2014, an increase of nearly three times after comparison [7]. As the largest number of people with diabetes worldwide [8], the overall prevalence of diabetes is 9.1% in China, with the highest prevalence of 65–74 years old accounting for 14.1% [9]. The high incidence of diabetes not only seriously affects individual's quality of life, but also imposes a heavy economic burden on the family, society, and country. According to the survey, China's medical expenses for diabetes in 2014 reached 80.33 billion yuan, and the per capita treatment cost was 2,188.73 yuan [10], which showed that a large amount of medical and health resources were consumed due to diabetes in China. The hypoglycemic effect of biomedicine has been proved; nevertheless, long-term or even lifelong medication is needed. For patients with advanced diabetes, who often accompany with diabetic complications, therefore, hypoglycemic agents in combination with drugs for complications are often used [1]. On this account, the cost of medication for diabetic patients is high. Even with the first-line hypoglycemic drug, metformin, there are side effects including lactic acidosis and digestive disorders [11, 12]. Therefore, it is urgent to seek treatment for diabetes with low cost and few side effects. After thousands of years of development and innovation, traditional Chinese medicine (TCM) has achieved a remarkable effect on treating diabetes based on its wholism and pattern identification and treatment at the advantages of low cost and few side effects [13]. TCM has become one of the major complementary and alternative medicines in East Asia, Europe, North America, and other regions [14].

However, due to the multicomponent and multitarget characteristics of TCM formulas, the action mechanism of most TCM formulas for diseases is still unclear. Therefore, we attempt to screen out the active components, effective targets, and functional pathways of drugs through network pharmacology and combined with the advantages of TCM classic formulas for the multitarget holistic treatment, to clarify the mechanism of TCM formulas and provide a systematic and clear idea for the treatment of T2DM.

T2DM belongs to “*xiāo kĕ* (wasting-thirst, 消渴)” and “splenic pure heat (*pí dān*, 脾瘅)” in TCM field. The cause of this disease is attributed to external contraction and internal damage. Externally, constraint of the six pathogenic factors results in the invasion of toxin, dryness-heat, and wind-heat pathogens into *zang-fu* organs, dryness generating, and liquid being damaged, which finally leads to *xiāo kĕ*. Internally, generation of heat because of emotional disorders and constraint of liver qi, damp-heat generated by intemperate eating, or yin depletion and abnormal exuberance of dryness-heat causes *xiāo kĕ*. In the early and middle stages of the onset, sturdy patients present with thirst, bitter taste in the mouth, halitosis, polydipsia, polyphagia, irritability, fullness and distension in the hypochondriac regions, dark urine, dry and hard stool, red tongue with yellow coating, and wiry, excess, and powerful pulse. According to pattern differentiation, these symptoms are diagnosed as liver-stomach heat pattern and its typical formula is Da-Chai-Hu Decoction (*Dà Chái Hú Tāng*, DCHD). The application of DCHD was first recorded in *Treatise on Febrile and Miscellaneous Diseases*. It has a history of more than 2,000 years. DCHD can harmonize *shaoyang* and drain heat bind in *yangming,* whose indication is overlap of diseases of *shaoyang* and *yangming.* DCHD is composed of Bupleurum, Scutellaria root, peony root, Pinellia Rhizome, immature bitter orange, rhubarb root and rhizome, fresh ginger, and Chinese date. Among them, Bupleurum resolves constraint and soothes the liver and rectifies qi when combined with peony root, one for dissipating and one for contraction. Bupleurum and Scutellaria root can clear liver heat, and immature bitter orange and rhubarb root and rhizome unblock the bowels and discharge heat in the stomach and intestines. Scutellaria root and rhubarb root and rhizome auxiliary with Pinellia Rhizome and fresh ginger open the middle *jiao*, with acrid herbs opening and bitter herbs promoting descent. Chinese date harmonizes the actions of all herbs in a formula. All herbs together can rectify qi to resolve constraint, clear heat, and dissipate masses so as to resolve constraint and discharge heat in the liver and stomach, and then *xiāo kĕ* itself resolves. The results of modern pharmacological experimental research show that the ingredients in DCHD can lower blood sugar. Bupleurum is the chief medicinal of DCHD. Jin et al showed that Bupleurum has the effect of treating diabetes [15]. Baicalin is the main active component of Scutellaria root. Experiment by Kuo et al. proved that the alcohol extract of DCHD can promote the absorption of glucose to reduce blood sugar, primarily by baicalin activating the IRS-1 and glut4 genes and the signaling cascade of 5′ AMP-activated protein kinase (AMPK), PI3K/Akt, and MAPK/ERK [16]. Naringin, the active component of immature bitter orange, combined with berberine can reduce liver lipid by activating AMPK and inhibiting Notch signaling pathway, thereby enhancing insulin sensitivity and improving resistance and remarkably improving oral glucose tolerance test (OGTT) [17]. Peony root can inhibit liver gluconeogenesis and lower blood sugar [18]. Rheum emodin, a medicinal component of rhubarb root and rhizome, can treat T2DM by inhibiting DPP4 protein [19]. Experimental studies have shown that ginger extract, ginger ketone, does have a therapeutic effect on diabetes. In addition, a randomized, double-blind, and placebo-controlled clinical trial has demonstrated that oral ginger supplementation can improve anthropomorphic data in diabetics by reducing the concentration of NF-*κ*B inflammatory factors [20].

**Figure 1:**
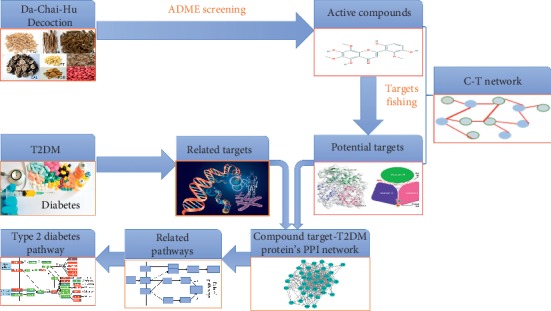
Flowchart of the systems pharmacology of DCHD in the treatment of D2TM.

Network pharmacology was first proposed by Hopkins in 2007. It is a discipline that studies the occurrence and development of diseases from the perspective of biological network, recognizes the interaction between drugs and the body, and guides the discovery of new drugs. T2DM is a complex metabolic disease determined by multiple genes and multiple factors, not by a single gene or a single factor. Therefore, complex diseases often cannot be effective by intervening in a single target, and the TCM formula is composed of various components, which can simultaneously act on multiple targets through multiple pathways [21]. Typical features of TCM treatment of diseases are “multipathway, multicomponent, multitarget,” which is consistent with most key ideas of network pharmacology and network biology and is able to treat complex diseases [22]. Network pharmacology aims to reveal the scientific basis of the traditional attributes of TCM. By establishing the molecular connection between TCM formulas and TCM patterns, it explores the action mechanism and the rules of herbal combination in formulas, thus laying the foundation for the development of new drugs. Through network pharmacology, we can not only explore the complex active molecular components and potential molecular targets in TCM formulas, but also understand the molecular relationships between components and components, and between components and complex disease in formulas, so that diseases can be targetedly treated [23]. Understanding the molecular relationship of TCM mentioned above not only provides sufficient theoretical support for TCM research but also enhances the acceptance of TCM worldwide [14]. Therefore, network pharmacology is of great significance to protect and develop TCM and promote the modernization of TCM. In recent years, with the rapid development of network pharmacology, more and more multitarget integrated control methods are used to predict the main active components and potential target groups of TCM formulas to determine their pharmacological mechanisms for a certain type of diseases [24]. For example, Mao et al. used network pharmacology to study the pharmacological mechanism of Xueshuan-Xinmai-Ning Tablet in the treatment of coronary heart disease [25]. Hu and Sun used network pharmacology and network topological algorithms to explore new TCM prescriptions for T2DM [26]. Dia et al. used network pharmacology to reveal the pharmacological and molecular mechanisms of Shen-Qi-Di-Huang Decoction in the treatment of diabetic nephropathy, which laid the foundation for further experimental research and expanded the rational application of Shen-Qi-Di-Huang Decoction in clinical practice [27]. Here, we also use the network pharmacology to reveal the pharmacological mechanism of DCHD in the treatment of T2DM from three aspects of active components, potential targets, and synthetic pathways (as shown in Figure 1).

## 2. Materials and Methods

### 2.1. Data Preparation

#### 2.1.1. Components of Each Herb in DCHD

**Figure 2:**
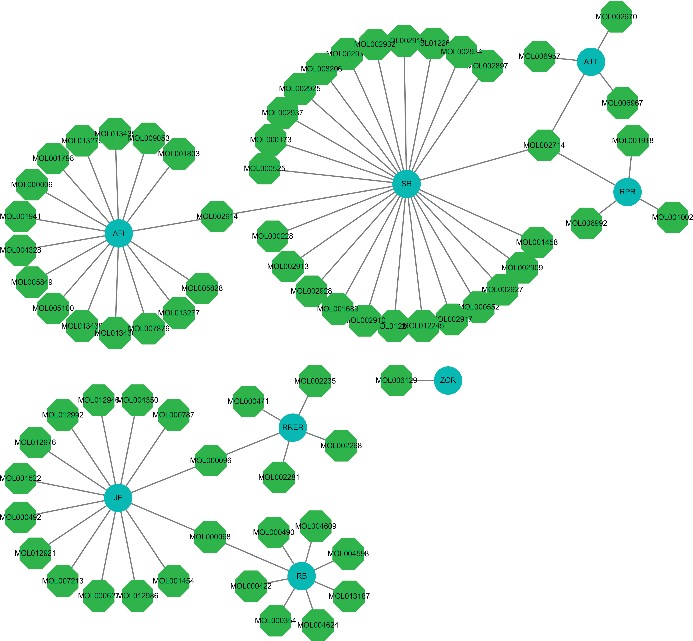
Herb-compound network.

Seventy-seven active components obtained from the eight herbs in DCHD were screened through a well-rounded literature search and the Traditional Chinese Medicine Systems Pharmacology Database (TCMSP, http://lsp.nwu.edu.cn/tcmsp.php) [28]. As a bran-new TCM research platform and database based on systematic pharmacology, TCMSP contains comprehensive contents, including active components, key absorption, distribution, metabolism, excretion (ADME) properties, drug-likeness (DL), active targets, pathways involved, and related diseases of all herbs included in Chinese Pharmacopoeia. From the TCMSP database, we obtained a total of 349 compounds, including 143 components in *Radix Bupleuri* (RB), 138 in *Scutellariae Radix* (SR), 119 in *Radix Paeoniae Rubra* (RPR), 116 in *Arum Ternatum Thunb* (ATT), 65 in *Aurantii Fructus Immaturus* (AFI), 92 in *Radix et Rhizoma Rhei* (RERR), 265 in *Zingiber Officinale Roscoe,* (ZOR), and 134 in *Jujubae Fructus* (JF). In order to screen out potential active compounds, the active molecules with high oral bioavailability (OB) in the organic metabolism from these components, we use the six principles of generic drugs of molecular weight (MW), AlogP, Hdon, Hacc, OB, and DL for screening in this work. From Table 1, the abovementioned six principles were used to screen the ingredients of all the herbs in DCHD, 77 potential components were finally obtained, including 8 in *Radix Bupleuri*, 25 in *Scutellariae Radix*, 4 in *Radix Paeoniae Rubra*, 4 in *Arum Ternatum Thunb*, 16 in *Aurantii Fructus Immaturus*, 5 in *Radix et Rhizoma Rhei*, 1 in *Zingiber Officinale Roscoe*, and 14 in *Jujubae Fructus* (as shown in Figure 2).

*(1) MW Prediction*. MW refers to the mass of a molecule. As one of the parameters of Lipinski's “rule of five,” it plays a critical role in pharmacological or biological activity because the size of MW will affect the size of the molecular fragment, then affecting the membrane absorption of drugs. Generally speaking, small molecules are more easily absorbed and distributed, while macromolecules are not only less easily absorbed, but also more easily excreted by bile. The components with MV from 180 to 500 Dalton are perceived as most available in drug therapy (MW ≤ 500).

*(2) AlogP Prediction*. The AlogP is the logarithm of the ratio of the equilibrium concentration of a compound in a nonaqueous phase to its equilibrium concentration in the aqueous phase in a neutral form. The logarithmic value of the partition coefficient *P* of the compound in octanol/water system is usually used as a measure of AlogP. Abiding by the Ghose–Crippen method, it is calculated from a regression equation based on the hydrophobicity contribution of 120 atom types, including common bonding of H, C, N, O, and S and the halogens (AlogP ≤5) [29].

*(3) Hdon and Hacc Prediction*. The hydrogen-bond capacity of a drug solute is an important parameter of permeability [30]. To cross the cell membrane, a drug molecule needs to break the hydrogen bonds formed with its aqueous environment [31]. Consequently, Hdon and Hacc negatively influence absorption, namely, these two parameters could influence the interaction of compounds and targets with the criteria of setting (Hdon ≤5 and Hacc ≤10).

*(4) OB Prediction*. OB is defined as the ratio of the number of active components absorbed into the circulatory system playing roles at the site of action to the sum of active components. OB is one of the most important indicators for evaluating ADME characteristics via bioinformatics. In this work, the OB screening was calculated by a powerful in-house system, OBioavail1.1 [32], and the compounds with OB ≥ 30% were filtered for further analysis. The following two basic sections describe the design principles of the threshold: (1) obtained information from the studied medicines using the compounds as little as possible in number and (2) elucidated the stability within reason by the reported pharmacological data [33].

*(5) DL Prediction*. DL means that a molecule contains some specific functional group or presents with the same or similar physical characteristics as most drugs. As a qualitative profile, DL is frequently used in drug design to evaluate whether a compound is chemically suitable for the drug, and how DL a molecule is with respect to parameters affecting its pharmacodynamic and pharmacokinetic profiles which will ultimately impact its ADME properties [34]. In this study, we performed a database-dependent model pre-DL (predicts drug-likeness) based on the molecular descriptors and Tanimoto coefficient. The DL of the compounds was calculated by the Tanimoto coefficient defined as follows:

(1) $$\begin{array}{c} T\left(A,B\right)=\frac{A\cdot B}{{\left\|A\right\|}^{2}+{\left\|B\right\|}^{2}-A\cdot B}. \end{array}$$

In this equation, *A* represents the descriptor of the new numerator and *B* indicates all the 6511 molecules selected from the DrugBank database (available online at http://www.drugbank.ca). The average of all descriptors was calculated by Dragon and the compounds with DL ≥ 0.18 were selected [35].

From the above, in order to obtain the core active components, the screening principle was defined as follows: MW ≤ 500, AlogP ≤5, Hdon ≤5, Hacc ≤10, OB ≥ 30%, and DL ≥ 0.18.

#### 2.1.2. Bioactive Component-Target Prediction for Each Herb in DCHD

After screening the active components, it was quite critical to find the targets of each component. First, in order to obtain putative targets of potential active components in DCHD, we searched for the corresponding targets based on the systematic drug targeting tool (SysDT) as described in our previous work. Second, we screened out drug targets corresponding to the active components that we screened before. Here, the principle of predicting the corresponding targets of pharmaceutical components was based on two mathematical approaches that effectively integrate large-scale chemical, genomic, and pharmacological information to accurately predict drug-target interactions [36], with a concordance rate of 82.83%, a sensitivity of 81.33%, and a specificity of 93.62%, respectively [28]. Next, the screened effective targets were combined with similar terms, i.e., the duplicates were removed to obtain the potential effective targets of all herbs in DCHD. Finally, we used the UniProtKB search function in the UniProt database (http://www.uniprot.org/) to obtain the official symbol for each protein by inputting the protein names with the species limited to “Homo sapiens.” UniProt database provides the scientific community with comprehensive, high-quality, and free access to protein sequence and functional information [27]. Eventually, 161 targets from DCHD were obtained without repeated targets of the same component (as shown in Table 2).

#### 2.1.3. T2DM-Specific Protein Collection

Information regarding T2DM-associated target genes was collected from the Comparative Toxicogenomics Database (CTD; http://ctdbase.org/), the Online Mendelian Inheritance in Man database (OMIM; https://www.ncbi.nlm.nih.gov/omim), and the Human Gene Database (http://www.genecards.org/). CTD is a robust, publicly available database that aims to advance understanding about how environmental exposures affect human health, which provides curated and inferred chemical-disease associations that are real associations extracted from the literature. All the targets of T2DM were deduced from the CTD database, and 200 targets were filtrated from the inference score of ≥57.56 [37]. OMIM is a comprehensive, authoritative compendium of human genes and genetic phenotypes that is freely available and updated regularly, emphasizing on the relationship between phenotype and genotype. OMIM is able to provide references for further research and tools for genomic analysis of cataloged genes [38]. GeneCards is a searchable, integrative database that provides comprehensive, user-friendly information on all annotated and predicted human genes. It automatically integrates gene-centric data from 125 web sources, including genomic, transcriptomic, proteomic, genetic, clinical, and functional information.

#### 2.1.4. Protein-Protein Interaction Data

All the protein-protein interaction (PPI) data were derived from STRING (https://string-db.org/, ver. 10.5) [39]. To be specific, the potential targets corresponding to the herbs in DCHD and those related to T2DM were imported into the STRING protein interaction database for PPI analysis with the organism defined as “Homo sapiens” and a confidence score of 0.7. The STRING database, updated on online, provides an open-source database and analysis tools for molecular interactions and aims to collect and integrate the information of all functional interactions between the expressed proteins by consolidating known and forecasted protein-protein association data for a large number of organisms [40].

### 2.2. Network Construction

#### 2.2.1. Network Construction

The network construction was performed as follows: (1) active compound-active compound target network of DCHD was constructed; (2) herb-compound target-T2DM target network was built by connecting the eight DCHD herbs with compound targets of each herb and T2DM targets; and (3) active compound targets-T2DM targets-other human proteins' PPI network was established.

We created the networks by utilizing the network visualization software Cytoscape (http://cytoscape.org/, ver. 3.5.1) [41]. Cytoscape is a graphic display network software for analysis of complex networks, visualization of biological pathways, and intermolecular interactions. Moreover, it also can integrate the complex networks, gene expression products, and other state data.

#### 2.2.2. Network Topological Features

There are three parameters used to evaluate every node in a network, including degree, node betweenness, and closeness. Degree indicates the number of edges between a node and other nodes in a network [42]. Betweenness evaluates the participation of a node in the shortest parts of the network and reflects the ability of nodes to proceed the rate of information flow in the network as well [43]. Closeness refers to the inverse of the sum of the distance from a node to other nodes [44]. The levels of the aforementioned three parameters reflect the importance of a node in the network. The higher the value of the parameter is, the more important the node becomes.

#### 2.2.3. Gene Ontology (GO) Enrichment and Pathway Analysis

The functional enrichment tool DAVID (DAVID Bioinformatics Resources, https://david.ncifcrf.gov/, ver. 6.8) [45] was used to calculate both the Kyoto Encyclopedia of Genes and Genomes pathway and GO biological processes enrichment.

## 3. Results and Discussion

### 3.1. Compound-Compound Target Network Analysis

**Figure 3:**
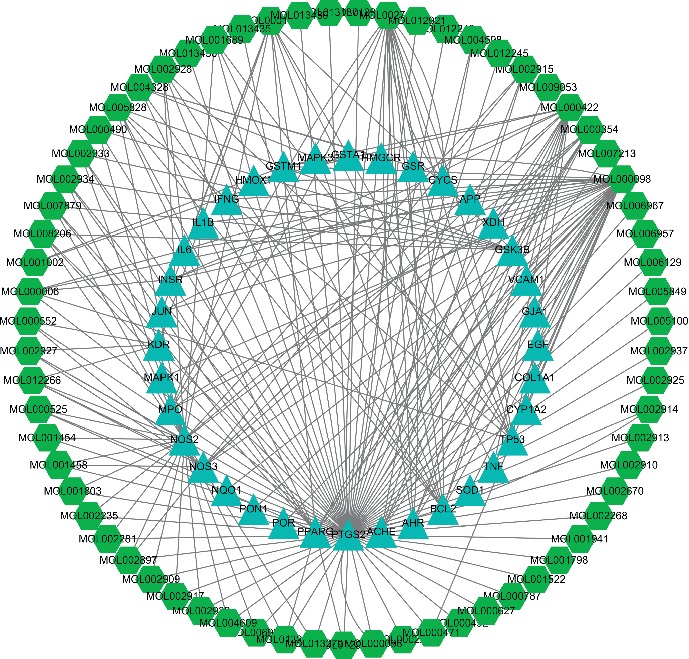
Target-compound network.

This network contained 238 nodes (161 compound target nodes and 77 active compound nodes), as shown in Figure 3. On the one hand, there were many compound targets corresponding to multiple components in the network, suggesting that different components often have common targets. The chief of all compounds is quercetin, which has the widest regulating range; in other words, the targets of quercetin are the maximum. On the other hand, some targets can be modulated by only one compound (peripheral nodes), such as TNF and CYP1A2 targets, while PTGS2 target can be controlled by all 77 compounds, which may be the pivotal targets in DCHD. Thus, from the network, we can have a general observation on the relationships between active compounds and targets from the compound-compound target network. The targets of all herbal composition in DCHD correspond to a variety of diseases besides T2DM, which can exert pharmacological effects on multiple diseases. Therefore, this network also fully reflects the characteristics of multicomponent-multitarget-multidisease of TCM formula.

### 3.2. T2DM Network Analysis

#### 3.2.1. T2DM PPI Network

**Figure 4:**
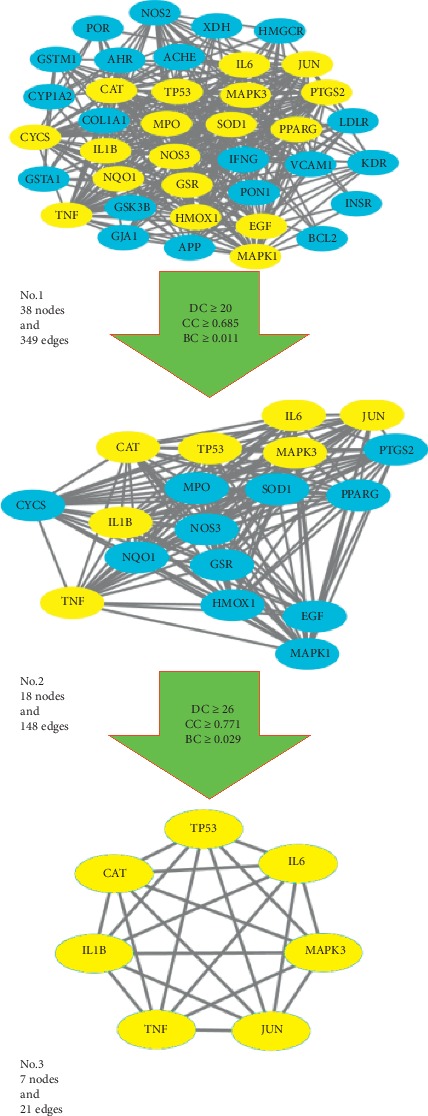
The process of topological screening for the PPI network. The yellow nodes represent the core targets, and the blue nodes represent the noncore targets.

The 161 targets of DCHD were corresponding to 200 targets of T2DM, and 38 repeat important targets were obtained (as shown in Table 3). In order to illuminate the significance of degree in compound targets, we created a PPI network about the relationship of the common targets between compounds and T2DM. This network was composed of 38 nodes and 349 edges. The more the edges between two nodes are, the greater the degree of association between the two targets exhibits. Then, a topological analysis was carried out to further screen the core targets with the critical effect (Figure 4). From the figure, No. 1 represented the interaction of 38 important targets, including the 18 yellow nodes representing the core targets obtained in the first topology analysis and the remaining 20 representing noncore targets. In the first topological analysis, based on the criteria of DC ≥ 20, CC ≥ 0.685, and BC ≥ 0.011, 18 nodes and 148 edges (No. 2) were obtained, and among the 18 nodes, 7 yellow nodes represent the core targets obtained in the second topology analysis, while 11 blue nodes represent the noncore targets. After the second topological analysis, with the criteria of DC ≥ 26, CC ≥ 0.771, and BC ≥ 0.029, PPI network (No. 3) was finally obtained, containing 7 nodes marked in yellow and 21 edges, indicating that there were 7 core targets obtained after topological analysis, namely, CAT, interleukin-6 (IL-6), tumor necrosis factor (TNF), IL-1*β*, JUN, MAPK3, and tumor protein 53 (TP53). The correlations of these 7 core targets were all over 27, of which the highest degrees were CAT and IL-6, up to 31, followed by TNF (30), and then to IL-1*β* (28), and to JUN, MAPK3, and TP53 (all 27). We found that DC was positively correlated with CC and BC. The larger the target of CC or BC is, the greater its DC value will be, indicating the powerful synergic effect between this target and other targets, as well as the critical role in the treatment of T2DM. With regard to CAT, the expression of catalase in organism can be enhanced by drugs to strengthen the antioxidant state and regulate the metabolic homeostasis and then effectively control blood sugar [46]. The degrees of proinflammatory cytokines and chemokines such as IL-6 and TNF are the same as or close to that of the antioxidant factors. Experiments show that drugs can inhibit the expression of inflammatory factors such as IL-6, TNF, IL-1*β*, c-JUN, and MAPK3 to reduce the proinflammatory response and thus to treat T2DM [47]. TP53 is a transcription factor that regulates the cell cycle. When TP53 transcriptional activity is activated, cell growth may be inhibited or in apoptosis, thereby effectively preventing cancer, but the activity of TP53 is closely related to the metabolism of the body. When metabolic disorders such as T2DM occur in human body, TP53 will increase in order to regulate the expression of apoptosis, proinflammatory, and metabolic genes. Therefore, TP53-mediated gene expression plays a vital role in the development of metabolic disorders in patients with T2DM [48, 49].

#### 3.2.2. Functional Analysis and Pathways of T2DM

In order to further clarify the biological functions of target genes *in vivo*, we systematically analyzed the biological processes related to 38 compound targets using Cytoscape plug-in CLUE-GO in a hierarchical manner [50].

**Figure 5:**
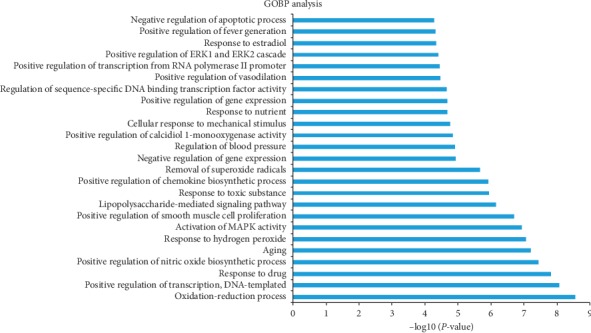
GO enrichment analysis of 30 nodes in biological process. “*Y*-axis” shows significantly enriched “biological process” related to target genes after GO analysis; “*X*-axis” represents the number of targets in −log10 (*P* value).

25 biological processes related to T2DM were mined in DAVID for specific pathway enrichment analysis. In Figure 5, most genes were mainly involved in several biological processes, including oxidation-reduction process, positive regulation of transcription, DNA-templated, positive regulation of nitric biosynthetic process, response to hydrogen peroxide, activation of MAPK activity, positive regulation of smooth muscle proliferation, and negative regulation of apoptotic process. Undoubtedly, these biological processes were all involved in the pathogenesis of T2DM, so they may serve as a potential therapeutic mechanism for T2DM.

Spanidis et al. evaluated the oxidative stress of type 2 diabetic patients by examining redox status markers and found that a redox state marker, namely, the redox potential in static state, in patients with T2DM was markedly increased compared with that in normal individuals. Therefore, the oxidative stress process may be one of the pathogenic mechanisms of diabetes mellitus [51]. Positive regulation of transcription can promote the synthesis of transcription factors such as TCF7L2 and mediate insulin secretion from islet *β*-cells, which is also a pathogenesis of T2DM [52]. Reynolds et al. found that decreased expression of endogenous nitric oxide synthase in obese T2DM patients may lead to a decrease in insulin-stimulated blood flow, thus positively regulating the synthesis of nitrogen-containing compounds may improve blood flow in patients with T2DM [53]. Koistinen et al. reported that the activation of p38MAPK enzyme in skeletal muscle of patients with T2DM was enhanced which is vital in the inflammatory process. Therefore, inhibition of p38MAPK enzyme activity could remarkably reduce the expression of inflammatory factors [54]. High-density lipoprotein in patients with T2DM causes abnormal proliferation of vascular smooth muscle cells due to abnormal components and modifications and mediates proinflammatory and atherogenic effects [55]. Interestingly, the abovementioned biological processes were mainly involved in apoptosis and inflammation. In terms of apoptosis, oxidative stress is an important link in apoptosis [56], especially in pancreatic cells, and due to its low antioxidant enzyme activity, it is susceptible to oxidative stress [57, 58]. MAPK is an important apoptosis signaling pathway. The apoptosis can be regulated by activating the MAPK cascade reaction pathways, in which p38MAPK can be activated by oxidative stress released by NADP oxidase, thereby promoting the occurrence of *β*-cell apoptosis [59]. Therefore, oxidative stress leading to the apoptosis of pancreatic *β*-cells serves as an important pathogenesis of T2DM. Endothelial nitric oxide synthase participates in the secretion of insulin-sensitizing substances in peripheral tissues, regulates insulin secretion and glucose tolerance, and inhibits *β*-cell apoptosis [60]. And the results of GO analysis also directly indicate that DCHD can inhibit the apoptosis of *β*-cells. With regard to inflammation, increase of p38MAPK enzyme activity and smooth muscle cell proliferation are both involved in the proinflammatory response in T2DM.

**Figure 6:**
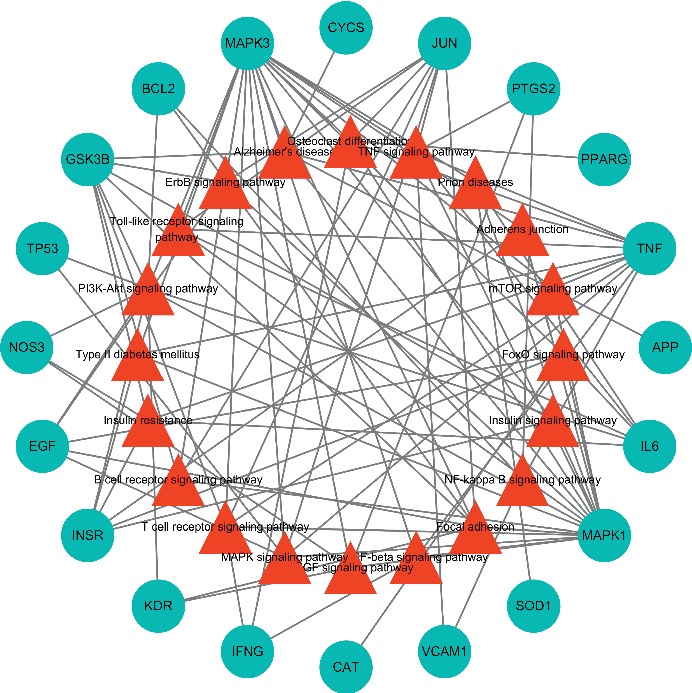
Target-pathway network. The red triangles represent the related pathways, and the blue round nodes represent the hub nodes.

The 38 core targets were imported into the DAVID, and 89 pathways were obtained. Through systematic review of relevant literature and summary of T2DM-related pathogenesis, 20 signal pathways closely related to T2DM were screened. To further integrate T2DM core targets, we finally detected two core pathways (Figure 6), namely, TNF signaling pathway and PI3K-AKT signaling pathway, which many important genes participate in. For instance, inflammatory factors such as IL-6, IL-1*β*, TNF-*α*, and vascular cell adhesion molecule (VCAM-1) all get involved in inflammatory response *via* TNF signaling pathway. Endothelial nitric oxide synthase (NOS3), glycogen synthase kinase-3*β* (GSK-3*β*), peroxisome proliferator antigen receptor-*γ* (PPAR-*γ*), and B-cell lymphoma-2 (Bcl-2) get involved in the regulation of apoptosis through PI3K/AKT signaling pathway. Therefore, the therapeutic mechanism of DCHD on T2DM mainly includes the following two modules: inflammation and cell apoptosis.

*(1) Inflammation Module*. T2DM is a low-grade inflammatory response disease [61]. The inflammatory reaction of T2DM is caused by metabolic disorders [62]. Due to overnutrition, adipocytes have accumulated too many nutrients in adipose tissues, liver, and skeletal muscle, which leads to the conversion of macrophages from anti-inflammatory “M2” (alternate activation) to a proinflammatory “M1” (classical activation) phenotype, inducing the increase of inflammatory factors [63]. Therefore, T2DM contains a large number of inflammatory factors triggering cascade reactions of inflammatory signals. This proinflammatory state in turn destructs the systemic insulin sensitivity and glucose homeostasis. As shown in Figure 6, TNFR1 is a member of TNF superfamily and is one of the main receptors of TNF. TNFR1 plays an important role in the positive regulation of NF-*κ*B signaling pathway mediated by inflammatory factors such as TNF-*α* and IL-1*β*. When there is no inflammatory factor stimulation in human body, NF-*κ*B binds to its inhibitor IĸB*α* in the cytoplasm when NF-*κ*B is not activated. For T2DM patients, inflammatory factors, growth factors, or chemokines can be activated in the body. In the presence of NF-*κ*B stimulation, inflammatory factors bind to TNFR1, and IKK*β* complexes are activated by NF-*κ*B-induced kinase phosphorylation, resulting in phosphorylation of IĸB*α* at the sites of Ser32 and Ser36, followed by degradation of the ubiquitin-proteasome pathway. This results in the dissociation of NF-*κ*B from its inhibitor IĸB*α*, exposing the nuclear localization sequence of NF-*κ*B. And then, NF-*κ*B is transferred to the nucleus to promote NF-*κ*B-dependent gene transcription. NF-*κ*B mainly regulates the transcriptional effects of IL-6, IL-1*β*, VCAM-1, COX-2, TNF-*α*, and JUN [64–67], thereby mediating the expression of proinflammatory factors to enhance inflammation.

**Figure 7:**
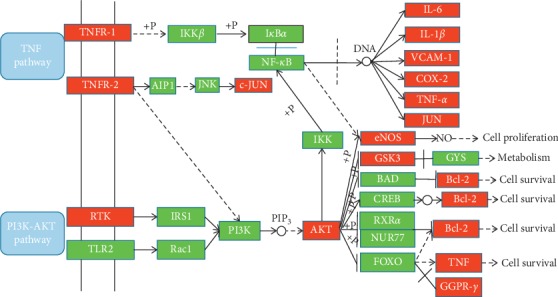
The T2DM pathway and therapeutic modules. The red rectangles represent the core targets, and the green rectangles represent the noncore targets.

*(2) Apoptosis Module*. The fundamental pathophysiological mechanism of T2DM is insulin resistance, which results in islet *β*-cell dysfunction and, in extreme cases, apoptosis of *β*-cells. Therefore, islet *β-*cell apoptosis serves as the main pathogenic feature of T2DM. Increasing evidence demonstrates that the PI3K/Akt signaling pathway plays a crucial role in regulating *β-*cell apoptosis [68]. PI3K is a class of phosphorylated phosphatidylinositol lipid kinase in the PI3K/Akt signaling pathway. AKT is a protein-serine/threonine kinase that is activated by the recruitment of phosphoinositide to the plasma membrane, also known as protein kinase B. According to the difference of serine/threonine residues, AKT is divided into three subtypes: AKT1, AKT2, and AKT3. AKT2 is mainly expressed in insulin-sensitive tissues such as skeletal muscle, adipose tissue, and liver. Therefore, the occurrence and development of AKT2 and T2DM are closely related. As shown in Figure 7, the process by which PI3K/Akt signaling pathway mediates islet *β*-cell apoptosis can be transduced by receptor tyrosine kinase (RTK) and TLR2 receptors. The first is the largest enzyme-linked receptor, which is both an enzyme and a cell surface receptor for cytokines, hormones including insulin, and polypeptide growth factors. The insulin receptor tyrosine kinase (IRTK) in RTKs is a transmembrane receptor composed of two extracellular *α*-subunits that bind to disulfides and two transmembrane *β*-subunits [69]. In insulin-sensitive tissues such as liver, adipose tissue, and muscle, insulin binds to the *α*-subunit of the IRTK, causing a conformational change in the RTK, thereby enhancing transphosphorylation between the transmembrane *β*-subunits further enhances the phosphokinase activity of the RTK, which in turn phosphorylates the insulin receptor substrate (IRS-1). IRS-1 successively combines with PI3K regulatory subunit p85 and catalytic subunit p110, and the activated PI3K produces the second messenger phospholipid inositol (3,4)-triphosphate to promote the activation of AKT. The other type is TLR2, which belongs to Toll-like receptor family and is a very conservative innate immune receptor that can recognize pathogen or injury-related molecular patterns, among which TLR2 is the member that recognizes pathogen-related molecular patterns most. When inflammatory factors in T2DM patients bind to TLR2 receptor, TLR2 activates Rac-1 [70], which belongs to the Rho family GTPase and is involved in the regulation of various cellular functions such as cytoskeletal reorganization, cell growth, and apoptosis [71]. Activated Rac-1 further activates PI3K [72] and AKT after PI3K rephosphorylation. After activation of AKT based on the above two methods, AKT phosphorylation can activate eNOS expression, and NO produced by eNOS can regulate systemic metabolism and insulin sensitivity [73]. In patients with T2DM, the insulin is relatively insufficient, and the NO produced via the PI3K/Akt signaling pathway is reduced, resulting in decreased insulin sensitivity, i.e., insulin resistance. Long-term insulin resistance results in islet *β*-cell dysfunction and ultimately *β*-cell apoptosis. AKT phosphorylation can also mediate glycogen synthesis by inhibiting GSK3 [62]. GSK3 is a serine/threonine kinase that inhibits glycogen synthesis by phosphorylating glycogen synthase (GS), destroying glucose homeostasis, facilitating the formation of insulin resistance, and possessing antiproliferation and *β*-cell apoptosis activity. Early studies have also shown that insulin inhibits GSK3 activity through phosphorylation induced via the PI3K/AKT signaling pathway and promotes glycogen synthesis and glucose utilization, thereby inhibiting apoptosis in *β*-cell cells [74]. In addition to the PI3K/Akt signaling pathway mentioned above, the TNF signaling pathway is also involved in the transduction of T2DM islet *β*-cell apoptosis. TNFR2 is a type II receptor of TNF-*α* in TNF signaling pathway, promoting cell migration, regeneration, and proliferation. TNFR2 activates JNK through its domain III binding to c-Jun-N-terminal kinase- (JNK-) specific signaling molecule AIP1. JNK is an important intracellular signal transduction pathway [75]. JNK activation promotes the expression of its downstream target gene c-JUN, which mediates the apoptosis of *β*-cells [76]. Targets for the regulation of *β*-cell apoptosis downstream of the PI3K/AKT signaling pathway include Bcl-2, TNF, and PPAR-*γ*. Bcl-2 is one of the oncogenes closely related to apoptosis [77]. A common feature of Bcl-2 family proteins is the presence of short conserved sequences consisting of 20 homologous (BH) motifs. There are three types of Bcl-2 proteins: prosurvival proteins, proapoptotic protein Bcl-2 homologous protein 3- (BH3-), and downstream multidomain proapoptotic effector proteins [78]. Many studies have shown that the intrinsic pathway of apoptosis is controlled by Bcl-2 family proteins present in the endoplasmic reticulum, mitochondria, and nuclear membrane [79–81]. Therefore, the occurrence of *β*-cell apoptosis is caused by the concentration of proapoptotic Bcl-2 protein in the *β*-cell exceeding the concentration of antiapoptotic protein on the mitochondrial membrane of the intrinsic pathway [82]. TNF is one of the cytokines involved in systemic inflammation produced by activated macrophages, and TNF-*α*-mediated inflammatory responses initiate the onset of islet *β*-cell apoptosis [83]. PPAR-*γ* is an important cell differentiation transcription factor, mainly expressed in adipose and skeletal muscle tissues. Activation of PPAR-*γ* in such mature cells can induce the expression of genes involved in the insulin signaling cascade, thereby improving insulin sensitivity and islet structure, and increase *β*-cells, and therefore, PPAR-*γ* has an effect of inhibiting *β*-cell apoptosis [84]. In summary, T2DM is a glucose metabolic disease characterized by *β*-cell apoptosis. Promotion of anti-*β*-cell apoptosis targets including Bcl-2 prosurvival protein and PPAR-*γ*, and inhibition of other pro-*β*-cell apoptosis targets such as TNF can inhibit *β*-cell apoptosis, thereby effectively controlling the development of T2DM.

In the present study, by network pharmacology, we deeply analyzed the two major pathological mechanisms of T2DM mediated by inflammation and apoptosis from the corresponding pathways of T2DM targets. Taking DCHD as an example, we explored the therapeutic effect of drugs on T2DM by blocking the corresponding inflammatory or apoptotic pathway leading to T2DM, which is of great significance in guiding the treatment of T2DM. However, the results obtained in this study have not been experimentally verified. Therefore, further experimental validation to increase the accuracy and feasibility of the research results is warranted.

## 4. Conclusion

As a traditionally prepared formula, DCHD is an important complementary and alternative medicine for the treatment of T2DM and has also been widely used in the treatment of T2DM in modern clinical practice. In this study, we attempted to clarify the relevant targets and related pathways for the treatment of T2DM with DCHD by integrating systematics, pharmacology, and genomics. Our research shows that the eight herbs in DCHD play a synergistic effect to some extent, mainly through the inflammatory signaling pathway represented by TNF signaling pathway and the apoptotic signaling pathway represented by PI3K/Akt signaling pathway. The targets in these two pathways may be the targets of T2DM. To sum up, network pharmacology established in this study preliminarily reveals the modern application value of TCM formula in multitarget treatment for related diseases at the molecular level and also provides a paradigm for studying multitarget treatment of diseases, which is of great practical significance.

## Figures and Tables

**Table 1:** 72 potential compounds of Da-Chai-Hu Decoction and their network parameters.

Mol ID	Molecule name	Structure	MW	AlogP	Hdon	Hacc	OB (%)	DL
MOL013187	Cubebin	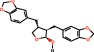	356.4	3.19	1	6	57.13	0.64
MOL004598	3,5,6,7-Tetramethoxy-2-(3,4,5-trimethoxyphenyl)chromone	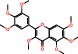	432.46	2.54	0	9	31.97	0.59
MOL004624	Longikaurin A	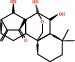	348.48	1.16	3	5	47.72	0.53
MOL004609	Areapillin	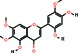	360.34	2.29	3	8	48.96	0.41
MOL000354	Isorhamnetin	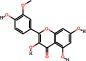	316.28	1.76	4	7	49.6	0.31
MOL000490	Petunidin	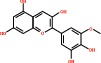	317.29	1.65	5	7	30.05	0.31
MOL000098	Quercetin	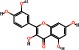	302.25	1.5	5	7	46.43	0.28
MOL000422	Kaempferol	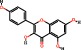	286.25	1.77	4	6	41.88	0.24
MOL001458	Coptisine	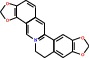	320.34	3.25	0	4	30.67	0.86
MOL002897	Epiberberine	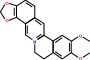	336.39	3.45	0	4	43.09	0.78
MOL002909	5,7,2,5-Tetrahydroxy-8,6-dimethoxyflavone	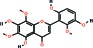	376.34	2.02	4	9	33.82	0.45
MOL002934	Neobaicalein	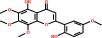	374.37	2.54	2	8	104.34	0.44
MOL002927	Skullcapflavone II	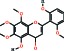	374.37	2.54	2	8	69.51	0.44
MOL012266	Rivularin	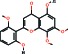	344.34	2.55	2	7	37.94	0.37
MOL000552	5,2′-Dihydroxy-6,7,8-trimethoxyflavone	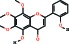	344.34	2.55	2	7	31.71	0.35
MOL002915	Salvigenin	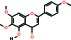	328.34	2.82	1	6	49.07	0.33
MOL002917	5,2′,6′-Trihydroxy-7,8-dimethoxyflavone	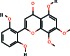	330.31	2.3	3	7	45.05	0.33
MOL002932	Panicolin	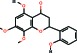	314.31	2.57	2	6	76.26	0.29
MOL012245	5,7,4′-Trihydroxy-6-methoxyflavanone	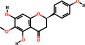	302.3	2.28	3	6	36.63	0.27
MOL002933	5,7,4′-Trihydroxy-8-methoxyflavone	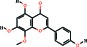	300.28	2.32	3	6	36.56	0.27
MOL012246	5,7,4′-Trihydroxy-8-methoxyflavanone	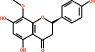	302.3	2.28	3	6	74.24	0.26
MOL008206	Moslosooflavone	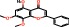	298.31	2.84	1	5	44.09	0.25
MOL002914	Eriodictyol (flavanone)	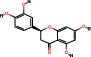	288.27	2.03	4	6	41.35	0.24
MOL002910	Carthamidin	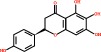	288.27	2.03	4	6	41.15	0.24
MOL002925	5,7,2′,6′-Tetrahydroxyflavone	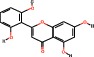	286.25	2.07	4	6	37.01	0.24
MOL001689	Acacetin	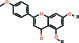	284.28	2.59	2	5	34.97	0.24
MOL002937	Dihydrooroxylin	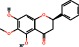	286.3	2.55	2	5	66.06	0.23
MOL002928	Oroxylin A	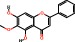	284.28	2.59	2	5	41.37	0.23
MOL000173	Wogonin	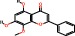	284.28	2.59	2	5	30.68	0.23
MOL002913	Dihydrobaicalin_qt	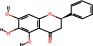	272.27	2.3	3	5	40.04	0.21
MOL000525	Norwogonin	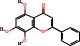	270.25	2.33	3	5	39.4	0.21
MOL002714	Baicalein	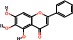	270.25	2.33	3	5	33.52	0.21
MOL000228	(2R)-7-Hydroxy-5-methoxy-2-phenylchroman-4-one	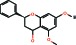	270.3	2.82	1	4	55.23	0.2
MOL001002	Ellagic acid	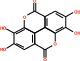	302.2	1.48	4	8	43.06	0.43
MOL001918	Paeoniflorgenone	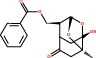	318.35	0.79	1	6	87.59	0.37
MOL006992	(2R,3R)-4-Methoxyl-distylin	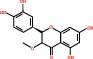	318.3	1.89	4	7	59.98	0.3
MOL002714	Baicalein	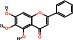	270.25	2.33	3	5	33.52	0.21
MOL002670	Cavidine	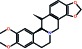	353.45	3.72	0	5	35.64	0.81
MOL006957	(3S,6S)-3-(Benzyl)-6-(4-hydroxybenzyl)piperazine-2,5-quinone	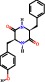	310.38	2.15	3	5	46.89	0.27
MOL006967	Beta-D-Ribofuranoside, xanthine-9	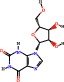	284.26	–1.29	5	9	44.72	0.21
MOL002714	Baicalein	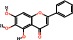	270.25	2.33	3	5	33.52	0.21
MOL005828	Nobiletin	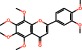	402.43	3.04	0	8	61.67	0.52
MOL001803	Sinensetin	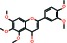	372.4	3.06	0	7	50.56	0.45
MOL013277	Isosinensetin	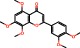	372.4	3.06	0	7	51.15	0.44
MOL009053	4-[(2S,3R)-5-[(E)-3-Hydroxyprop-1-enyl]-7-methoxy-3-methylol-2,3-dihydrobenzofuran-2-yl]-2-methoxy-phenol	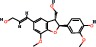	358.42	2.16	3	6	50.76	0.39
MOL007879	Tetramethoxyluteolin	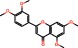	342.37	3.07	0	6	43.68	0.37
MOL013435	Poncimarin	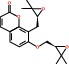	330.41	2.74	0	5	63.62	0.35
MOL013436	Isoponcimarin	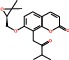	330.41	2.94	0	5	63.28	0.31
MOL013279	5,7,4′-Trimethylapigenin	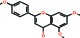	312.34	3.09	0	5	39.83	0.3
MOL013430	Prangenin	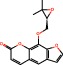	286.3	2.49	0	5	43.6	0.29
MOL001798	Neohesperidin_qt	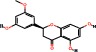	302.3	2.28	3	6	71.17	0.27
MOL005100	5,7-Dihydroxy-2-(3-hydroxy-4-methoxyphenyl)chroman-4-one	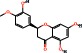	302.3	2.28	3	6	47.74	0.27
MOL000006	Luteolin	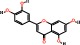	286.25	2.07	4	6	36.16	0.25
MOL002914	Eriodictyol (flavanone)	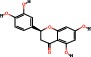	288.27	2.03	4	6	41.35	0.24
MOL005849	Didymin	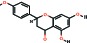	286.3	2.55	2	5	38.55	0.24
MOL001941	Ammidin	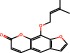	270.3	3.65	0	4	34.55	0.22
MOL004328	Naringenin	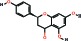	272.27	2.3	3	5	59.29	0.21
MOL002235	Eupatin	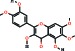	360.34	1.99	3	8	50.8	0.41
MOL002268	Rhein	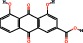	284.23	1.88	3	6	47.07	0.28
MOL000471	Aloe-emodin	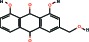	270.25	1.67	3	5	83.38	0.24
MOL000096	(−)-Catechin	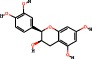	290.29	1.92	5	6	49.68	0.24
MOL002281	Toralactone	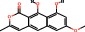	272.27	2.25	2	5	46.46	0.24
MOL006129	6-Methylgingediacetate2	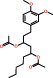	394.56	4.55	0	6	48.73	0.32
MOL000787	Fumarine	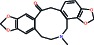	353.4	2.95	0	6	59.26	0.83
MOL001454	Berberine	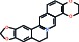	336.39	3.45	0	4	36.86	0.78
MOL004350	Ruvoside_qt	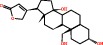	390.57	2.29	3	5	36.12	0.76
MOL012986	Jujubasaponin V_qt	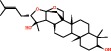	472.78	4.65	2	4	36.99	0.63
MOL012946	Ziziphus saponin I_qt	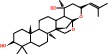	472.78	4.39	2	4	32.69	0.62
MOL000627	Stepholidine	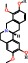	327.41	3.1	2	5	33.11	0.54
MOL012992	Mauritine D	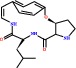	342.46	1.62	2	6	89.13	0.45
MOL007213	Nuciferine	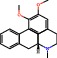	295.41	3.57	0	3	34.43	0.4
MOL012976	Coumestrol	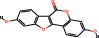	268.23	3.01	2	5	32.49	0.34
MOL012921	Stepharine	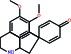	297.38	1.82	1	4	31.55	0.33
MOL001522	(S)-coclaurine	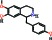	285.37	2.83	3	4	42.35	0.24
MOL000098	Quercetin	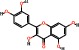	302.25	1.5	5	7	46.43	0.28
MOL000492	(+)-Catechin	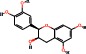	290.29	1.92	5	6	54.83	0.24
MOL000096	(−)-Catechin	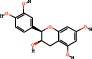	290.29	1.92	5	6	49.68	0.24

**Table 2:** The Da-Chai-Hu Decoction-related target information.

UniProt ID	Target name	Gene name	Drug
P23219	Prostaglandin G/H synthase 1	PTGS1	RB/SR/RPR/ATT/RRER/AFI/JF
P00742	Coagulation factor Xa	F10	RB/SR/ATT/AFI/RRER/ZOR/JF
P35354	Prostaglandin G/H synthase 2	PTGS2	RB/SR/RPR/ATT/RRER/AFI/ZOR/JF
P07550	Beta-2 adrenergic receptor	ADRB2	RB/SR/ATT/AFI/JF
P08238	Heat-shock protein HSP 90	HSP90AB1	RB/SR/RPR/ATT/RRER/AFI/JF
P00734	Thrombin	F2	RB/SR/AFI/RRER/JF
Q99527	Estrogen receptor	GPER1	RB/SR/RPR/RRER/AFI/ZOR/JF
P10275	Androgen receptor	AR	RB/SR/RPR/ATT/AFI/RRER/JF
P08709	Coagulation factor VII	F7	RB/SR/ATT/AFI/RRER/JF
P22303	Acetylcholinesterase	ACHE	RB/SR/AFI/JF
P11388	DNA topoisomerase II	TOP2	RB/SR/ATT/AFI/RRER/JF
Q92731	Estrogen receptor-beta	ESTRB	RB/SR/AFI/RRER
P07477	Trypsin-1	PRSS1	RB/SR/RPR/ATT/RRER/AFI/JF
Q15596	Nuclear receptor coactivator 2	NCOA2	RB/SR/RPR/ATT/RRER/AFI/JF
P0DP23	Calmodulin	CALM	RB/SR/RPR/ATT/RRER/AFI/ZOR/JF
P11229	Muscarinic acetylcholine receptor M1	CHRM1	RB/SR/ATT/AFI/JF
P47869	Gamma-aminobutyric-acid receptor alpha-2 subunit	GABRA2	RB
P34903	Gamma-aminobutyric-acid receptor alpha-3 subunit	GABRA3	RB/JF
P08172	Muscarinic acetylcholine receptor M2	CHRM2	RB/JF
Q16445	Gamma-aminobutyric-acid receptor subunit alpha-6	GABRA6	RB
P35228	Nitric oxide synthase, inducible	NOS2	RB/SR/RPR/RRER/AFI/JF
Q14524	Sodium channel protein type 5 subunit alpha	SCN5A	RB/SR/ATT/RRER/AFI/JF
P18031	mRNA of protein-tyrosine phosphatase, nonreceptor type 1	PTPN1	RB/SR/AFI
P27487	Dipeptidyl peptidase IV	DPP4	RB/SR/RPR/ATT/RRER/AFI/JF
P01857	Ig gamma-1 chain C region	IGHG1	RB/SR/RRER
Q15788	Nuclear receptor coactivator 1	NCOA1	RB/SR/RPR/ATT/AFI/JF
P37231	Peroxisome proliferator-activated receptor-gamma	PPARG	RB/SR/AFI/JF
Q16539	Mitogen-activated protein kinase 14	MAPK14	RB/SR/AFI
P49841	Glycogen synthase kinase-3 beta	GSK3B	RB/SR/AFI
P24941	Cell division protein kinase 2	CDK2	RB/SR/AFI
P48736	Phosphatidylinositol-4,5-bisphosphate 3-kinase catalytic subunit, gamma isoform	PIK3CG	RB/SR/RPR/ATT/RRER/AFI/JF
P17612	mRNA of PKA catalytic subunit C-alpha	PRKACA	RB/SR/RPR/ATT/RRER/AFI/JF
P11309	Proto-oncogene serine/threonine-protein kinase Pim-1	PIM1	RB/AFI
P20248	Cyclin-A2	CCNA2	RB
P11217	Glycogen phosphorylase, muscle form	PYGM	RB/SR/AFI
Q03181	Peroxisome proliferator-activated receptor delta	PPARD	RB/SR/RRER
O14757	Serine/threonine-protein kinase Chk1	CHEK1	RB/SR/AFI/RRER
P15121	Aldose reductase	AKR1B1	RB/RRER/JF
P29474	Nitric oxide synthase, endothelial	NOS3	RB/SR/AFI/JF
P14867	Gamma-aminobutyric acid receptor subunit alpha-1	GABRA1	RB/SR/RPR/AFI/JF
P27338	Amine oxidase (flavin-containing) B	MAOB	RB/SR/AFI/JF
P42262	Glutamate receptor 2	GRIA2	RB
P47989	Xanthine dehydrogenase/oxidase	XDH	RB/AFI/JF
P06401	Progesterone receptor	PGR	RB/RPR
P23975	Sodium-dependent noradrenaline transporter	SLC6A2	RB/JF
P35368	Alpha-1B adrenergic receptor	ADRA1B	RB/SR/ATT/AFI/JF
P10415	Apoptosis regulator Bcl-2	BCL2	RB/SR/RPR/ATT/AFI/JF
P01375	Tumor necrosis factor	TNF	RB/SR/AFI/RRER/JF
P05412	Transcription factor AP-1	JUN	RB/SR/AFI/RRER/JF
P45983	Mitogen-activated protein kinase 8	MAPK8	RB/AFI
P03956	Interstitial collagenase	MMP1	RB/SR/AFI/JF
P06493	Cell division control protein 2 homolog	CDC2	RB/SR/RPR/ATT/RRER/JF
P09601	Heme oxygenase 1	HMOX1	RB/AFI/JF
P08684	Cytochrome P450 3A4	CYP3A4	RB/JF
P05177	Cytochrome P450 1A2	CYP1A2	RB/SR/JF
P16581	E-selectin	SELE	RB/JF
P19320	Vascular cell adhesion protein 1	VCAM1	RB/JF
P09917	Arachidonate 5-lipoxygenase	ALOX5	RB/JF
P09211	Glutathione S-transferase P	GSTP1	RB/RPR/AFI/JF
P35869	Aryl hydrocarbon receptor	AHR	RB/SR/RPR/ATT/JF
P06213	Insulin receptor	INSR	RB/AFI/JF
Q08209	Serine/threonine-protein phosphatase 2B catalytic subunit alpha isoform	PPP3CA	RB
P09488	Glutathione S-transferase Mu 1	GSTM1	RB/RPR/JF
P28161	Glutathione S-transferase Mu 2	GSTM2	RB/RPR/JF
P42330	Aldo-keto reductase family 1 member C3	AKR1C3	RB
Q12809	Potassium voltage-gated channel subfamily H member 2	KCNH2	RB/SR/ATT/AFI/JF
P08254	Stromelysin-1	MMP3	RB/JF
P19793	Retinoic acid receptor RXR-alpha	RXRA	RB/SR/ATT/AFI/JF
P00533	Epidermal growth factor receptor	EGFR	RB/AFI/JF
P15692	Vascular endothelial growth factor A	VEGFA	RB/SR/ATT/RRER/AFI/JF
P00749	Urokinase-type plasminogen activator	PLAU	RB/JF
P08253	72 kDa type IV collagenase	MMP2	RB/RPR/AFI/JF
P28482	Mitogen-activated protein kinase 1	MAPK1	RB/SR/AFI/JF
P01133	Proepidermal growth factor	EGF	RB/JF
P06400	Retinoblastoma-associated protein	RB1	RB/AFI/JF
P05231	Interleukin-6	IL6	RB/SR/AFI/JF
P04637	Cellular tumor antigen p53	TP53	RB/SR/RPR/ATT/RRER/AFI/JF
P16435	NADPH-cytochrome P450 reductase	POR	RB/JF
P11926	Ornithine decarboxylase	ODC1	RB/JF
P11387	DNA topoisomerase 1	TOP1	RB/AFI/JF
P00441	Superoxide dismutase (Cu-Zn)	SOD1	RB/AFI/JF
P11021	78 kDa glucose-regulated protein	HSPA5	RB/JF
Q13085	Acetyl-CoA carboxylase 1	ACACA	RB/JF
P13726	Tissue factor	F3	RB/JF
P17302	Gap junction alpha-1 protein	GJA1	RB/JF
P01584	Interleukin-1 beta	IL1B	RB/RRER/JF
Q99616	C-C motif chemokine 2	CCL13	RB/SR/JF
P43115	Prostaglandin E2 receptor EP3 subtype	PTGER3	RB/SR/JF
P49888	Estrogen sulfotransferase	SULT1E1	RB/SR/JF
O43451	Maltase-glucoamylase, intestinal	MGAM	RB/SR/JF
P60568	Interleukin-2	IL2	RB/AFI/JF
P00750	Tissue-type plasminogen activator	PLAT	RB/JF
P07204	Thrombomodulin	THBD	RB/JF
P02452	Collagen alpha-1(I) chain	COL1A1	RB/JF
P01579	Interferon-gamma	IFNG	RB/AFI/JF
P05164	Myeloperoxidase	MPO	RB/SR/RPR/ATT/JF
P15559	NAD(P)H dehydrogenase [quinone] 1	NQO1	RB/JF
P02461	Collagen alpha-1(III) chain	COL3A1	RB/JF
P15309	Prostatic acid phosphatase	ACPP	RB/JF
P07339	Cathepsin D	CTSD	RB/JF
P27169	Serum paraoxonase/arylesterase 1	PON1	RB/JF
Q9Y233	cAMP and cAMP-inhibited cGMP 3′,5′-cyclic phosphodiesterase 10A	PDE10A	SR/ATT/JF
Q12791	Calcium-activated potassium channel subunit alpha 1	KCNMA1	SR/AFI
P35968	Vascular endothelial growth factor receptor 2	KDR	SR/RRER/JF
P54289	Voltage-dependent calcium channel subunit alpha-2/delta-1	CACNA2D1	SR
Q14432	CGMP-inhibited 3′,5′-cyclic phosphodiesterase A	PDE3A	SR/RPR/ATT/AFI/JF
P61925	cAMP-dependent protein kinase inhibitor alpha	PKIA	SR/RRER
P50613	Cell division protein kinase 7	CDK7	SR
P11712	Cytochrome P450 2C9	CYP2C9	SR
P99999	Cytochrome c	CYCS	SR/RPR/ATT
Q9GZT9	Egl nine homolog 1	EGLN1	SR/RPR/ATT
P00918	Carbonic anhydrase II	CA2	SR/AFI/ATT
P36544	Neuronal acetylcholine receptor protein, alpha-7 chain	CHRNA7	SR/JF
P49327	Fatty acid synthase	FASN	SR/AFI/RRER/JF
P21728	Dopamine D1 receptor	DRD1	SR/ATT/JF
P20309	Muscarinic acetylcholine receptor M3	CHRM3	SR/ATT/JF
P35348	Alpha-1A adrenergic receptor	ADRA1A	SR/JF
Q01959	Sodium-dependent dopamine transporter	SLC6A3	SR/JF
P31645	Sodium-dependent serotonin transporter	SLC6A4	SR/ATT/JF
P08263	Glutathione S-transferase A1	GSTA1	RPR
P09210	Glutathione S-transferase A2	GSTA2	RPR
P08588	Beta-1 adrenergic receptor	ADRB1	ATT
P08912	Muscarinic acetylcholine receptor M5	CHRM5	ATT/JF
P46098	5-Hydroxytryptamine receptor 3A	HTR3A	ATT/JF
P18825	Alpha-2C adrenergic receptor	ADRA2C	ATT/AFI/JF
P08173	Muscarinic acetylcholine receptor M4	CHRM4	ATT/JF
P41143	Delta-type opioid receptor	OPRD1	ATT/JF
P28223	5-Hydroxytryptamine 2A receptor	HTR2A	ATT/JF
P28335	5-Hydroxytryptamine 2C receptor	HTR2C	ATT/JF
P25100	Alpha-1D adrenergic receptor	ADRA1D	ATT/JF
P35372	Mu-type opioid receptor	OPRM1	ATT/JF
P28702	Retinoic acid receptor RXR-beta	RXRB	ATT
P00491	Purine nucleoside phosphorylase	PNP	ATT
P47712	Cytosolic phospholipase A2	PLA2G4A	AFI
Q09428	Sulfonylurea receptor 1	ABCC8	AFI
P56817	Beta-secretase	BACE1	AFI
P11802	Cell division protein kinase 4	CDK4	AFI
P05067	Amyloid beta A4 protein	APP	AFI
P55210	Caspase-7	CASP7	AFI
P08581	Hepatocyte growth factor receptor	MET	AFI
P27361	Mitogen-activated protein kinase 3	MAPK3	AFI
P01130	Low-density lipoprotein receptor	LDLR	AFI
P04040	Catalase	CAT	AFI/JF
P04035	3-Hydroxy-3-methylglutaryl-coenzyme A reductase	HMGCR	AFI
P00390	Glutathione reductase, mitochondrial	GSR	AFI
P33527	Multidrug resistance-associated protein 1	ABCC1	AFI
Q04828	Aldo-keto reductase family 1 member C1	AKR1C1	AFI
P17174	Aspartate aminotransferase, cytoplasmic	GOT1	AFI
P80404	4-Aminobutyrate aminotransferase, mitochondrial	ABAT	AFI
P35610	Sterol O-acyltransferase 1	SOAT1	AFI
Q13698	Voltage-dependent L-type calcium channel subunit alpha-1S	CACNA1S	JF
P27815	Type IV phosphodiesterase	PDE4A	JF
P08235	Mineralocorticoid receptor	NR3C2	JF
P21918	D(1B) dopamine receptor	DRD5	JF
P21917	D(4) dopamine receptor	DRD4	JF
P18089	Alpha-2B adrenergic receptor	ADRA2B	JF
Q15822	Neuronal acetylcholine receptor subunit alpha-2	CHRNA2	JF
P14416	D(2) dopamine receptor	DRD2	JF
P20813	Cytochrome P450 2B6	CYP2B6	JF
P08913	Alpha-2A adrenergic receptor	ADRA2A	JF
O75469	Pregnane X receptor	NR1I2	AFI

**Table 3:** The related targets' information of D-D.

Number	Gene name
1	PTGS2
2	ACHE
3	NOS2
4	PPARG
5	GSK3B
6	NOS3
7	BCL2
8	TNF
9	JUN
10	HMOX1
11	CYP1A2
12	VCAM1
13	AHR
14	INSR
15	GSTM1
16	MAPK1
17	EGF
18	IL6
19	TP53
20	POR
21	SOD1
22	GJA1
23	IL1B
24	COL1A1
25	IFNG
26	MPO
27	NQO1
28	PON1
29	KDR
30	CYCS
31	GSTA1
32	APP
33	MAPK3
34	LDLR
35	CAT
36	HMGCR
37	GSR
38	XDH

## Data Availability

The data used to support the findings of this study are available from the corresponding author upon request.

## Abbreviations

DCHD: Da-Chai-Hu Decoction
TCM: Traditional Chinese medicine
OB: Oral bioavailability
TCMSP: Traditional Chinese Medicine Systems Pharmacology Analysis Platform
DL: Drug-likeness
PPI: Protein-protein interaction
DAVID: Database for Annotation, Visualization, and Integrated discovery
GO: Gene ontology
BP: Biological process
CC: Cellular component
MF: Molecular function
MW: Molecular weight
KEGG: Kyoto Encyclopedia of Genes and Genomes
TTD: Therapeutic Targets Database
DC: Degree centrality
BC: Betweenness centrality
CC: Closeness centrality
OGTT: Oral glucose tolerance test
SysDT: Systematic drug targeting tool.

## Supplementary Materials

Supplementary Materials 1ingredients: the 77 potential Da-Chai-Hu ingredients obtained through six principles of screening. 2Drug-related targets: related targets obtained for each drug ingredient. 3Drug-targets: drug targets based on the total drug targets obtained in 2Drug-related targets, the duplicates are deleted, and the corresponding specifications are found for each target obtained. 4Disease-targets: all the targets related to T2DM, and the top 200 targets taken in order from the largest to the smallest according to the inference score. 5Common targets of disease and drug: a duplicate of the drug's total targets and disease targets, which is a common target for drugs and diseases. 6Topology: screened twice by “degree, closeness centrality, and betweeness” to get the final 7 core targets. 7GO-BP: 25 biological processes related to T2DM mined in DAVID for specific pathway enrichment analysis. 8 PPI: the 161 targets of DCHD corresponding to 200 targets of T2DM, and 38 repeat important targets obtained. In order to illuminate the significance of degree in compound targets, we created a PPI network about the relationship of the common targets between compounds and T2DM. This network was composed of 38 nodes and 349 edges. C-T.cys: Targets-compounds network. Figure 3. D-C.cys: herbs-compounds network. Figure 2 ppi.cys: interrelationship between 38 targets. Figure 4. T-P.cys: target-pathway network. Figure 6.
